# Association of Metformin with the Risk of Dementia: A Population-Based Retrospective Cohort Study in Taiwan

**DOI:** 10.3390/healthcare13131537

**Published:** 2025-06-27

**Authors:** Zhong-Bao Hou, Yu-Ching Chou, Tsan Yang, Chien-An Sun

**Affiliations:** 1 Department of Public Health, Fu Jen Catholic University, New Taipei City 24205, Taiwan; 412446197@m365.fju.edu.tw; 2 School of Public Health, National Defense Medical Center, Taipei City 11490, Taiwan; trishow@mail.ndmctsgh.edu.tw; 3 Master Program in Transdisciplinary Long-Term Care, Neipu, Pingtung County 91202, Taiwan; san.yang@msa.hinet.net

**Keywords:** cohort study, dementia, metformin, type 2 diabetes

## Abstract

**Background**: Diabetes is rapidly increasing in developing and industrializing nations, primarily due to type 2 diabetes (T2DM). With the global prevalence of diabetes steadily increasing, estimates suggest that by 2045, nearly 548 million people will be living with the disease worldwide. Alzheimer’s disease (AD), recognized as the primary contributor to dementia in aging populations, exhibits an escalating prevalence that parallels the demographic shifts toward older age groups worldwide. This progressive neurodegenerative disorder has emerged as a critical public health challenge, with epidemiological patterns closely tracking the trajectory of population aging across industrialized and developing nations. This study investigates whether metformin may help reduce the risk of dementia. Previous studies from various countries have explored the association between metformin use and dementia risk; however, the findings have been inconsistent. Therefore, we conducted this study to examine whether the observed protective effect of metformin also applies to the Taiwanese (Han Chinese) population, potentially providing valuable insights into ethnic or regional differences in drug response. **Methods**: We conducted a retrospective cohort study using data from the Longitudinal Health Insurance Database 2000 (LHID2000), including 2 million individuals from 2000 to 2013. Patients with T2DM aged ≥40 years who initiated metformin between 2000 and 2005 formed the exposed group, while those starting other second-line antidiabetic medications formed the non-exposed group. Propensity score matching was used to control for age, sex, index date, and major comorbidities. Incident dementia (2007–2013) was identified using relevant ICD-9-CM codes. Adjusted hazard ratios were estimated using Cox regression with time-dependent covariates. **Results**: The metformin-exposed cohort demonstrated a risk reduction for dementia incidence relative to the comparator group (adjusted HR 0.472, 95% CI = 0.328–0.679). This protective association remained robust in sex-stratified analyses and age-stratified subgroups. Temporal analysis further revealed a duration-dependent risk attenuation, with extended therapeutic exposure correlating with progressive dementia risk decrement. **Conclusions**: Our findings suggest that metformin use may be associated with a lower risk of developing dementia in individuals with type 2 diabetes mellitus.

## 1. Introduction

### Background

Industrialized nations are experiencing an accelerating trajectory in diabetes epidemiological burden. Current epidemiological models project 190 million individuals globally affected by diabetes, with surveillance data anticipating a rise to 330 million cases by 2025—predominantly attributed to type 2 diabetes mellitus (T2DM) [1]. Longitudinal projections further estimate a 2045 global disease prevalence exceeding 600 million, representing a 150% increase relative to 2020 baseline figures [2]. According to the National Health Research Institutes’ 2019 Diabetes Yearbook in Taiwan, approximately 2.3 million people in Taiwan have diabetes, and this number continues to increase at a rate of 25,000 individuals per year. The prevalence rate of diabetes among Taiwanese adults aged 18 and above is 11.1%, and in the 2021 national top 10 causes of death statistics, diabetes ranks fifth, causing nearly 10,000 deaths annually [3,4]. Clinically, if diabetes is not treated, it may lead to various complications. In clinical settings, inadequately managed diabetes mellitus predisposes patients to multilevel pathophysiological sequelae. Acute metabolic derangements manifest as diabetic ketoacidosis and hyperosmolar hyperglycemic state [5], whereas chronic disease progression elevates risks for macroangiopathic sequelae (cardiovascular events, cerebrovascular accidents) and microvascular pathologies (chronic renal impairment, lower extremity complications, retinal vasculopathy). In summary, diabetes requires timely detection and initiation of treatment to prevent it from leading to serious complications.

Alzheimer’s disease (AD) is a progressive neurodegenerative disorder characterized by memory loss and cognitive decline, and it is the most common form of dementia in older adults [6,7]. Its prevalence is rising with an aging global population [8]. Epidemiological studies consistently report a strong association between T2DM and AD [7,9,10], implying shared underlying mechanisms [11]. In fact, T2DM is associated with about a 60% increased risk of developing AD [9,11]; diabetic individuals show roughly 1.6-fold higher rates of dementia than non-diabetics [12]. This link may reflect common pathophysiological factors such as insulin resistance, impaired glucose metabolism, inflammation, oxidative stress, and accelerated aging [11]. Accordingly, therapies that can address both metabolic and cognitive aspects of these diseases are of considerable clinical interest.

Previous studies have demonstrated a correlation between hyperglycemia and cognitive decline [9,11]. Prolonged hyperglycemia can cause cerebrovascular damage, impairing cognitive function and increasing the risk of dementia. Insulin resistance, a hallmark of T2DM, is also considered a critical factor in AD pathogenesis [9,13]. Impaired insulin signaling can disrupt energy metabolism in brain cells, leading to β-amyloid deposition and tau protein hyperphosphorylation—two hallmark pathological features of AD [9,11]. Furthermore, other studies suggest that insulin resistance and deficiency may interact with amyloid-β and tau protein phosphorylation [9]. The abnormal deposition and aggregation of these proteins contribute to neuronal damage and dysfunction. Additionally, both diabetes and AD are associated with chronic inflammatory responses [7,11,14]. The release of inflammatory factors can exacerbate neurodegeneration and promote disease progression. Given the strong correlation between T2DM and dementia, identifying medications that can simultaneously address both conditions is of significant clinical value.

The therapeutic algorithm for diabetes mellitus prioritizes metformin as the foundational pharmacotherapy, with subsequent pharmacotherapeutic options stratified into insulin preparations and oral antihyperglycemic agents. The latter category encompasses sulfonylureas, dipeptidyl peptidase-4 (DPP-4) inhibitors, meglitinide derivatives, thiazolidinedione compounds, and α-glucosidase enzymatic modulators. Metformin, a widely used antidiabetic medication, has been the cornerstone of T2DM management for many years due to its high safety profile, minimal side effects, and low cost. Recently, metformin has garnered attention for its potential neuroprotective effects demonstrated in animal models. Metformin may exert its therapeutic effects on AD by regulating glucose metabolism, reducing amyloid plaque deposition, inhibiting tau protein phosphorylation, and promoting autophagy [11]. However, clinical trial results have been inconsistent, and their efficacy in individuals with mild cognitive impairment or early-stage AD remains unclear [11,15]. This research utilizes extensive datasets from the NHIRD to examine the potential neuroprotective properties of metformin in dementia prevention among Taiwanese individuals diagnosed with T2DM.

Diabetes mellitus is characterized as a chronic metabolic disorder manifested through sustained hyperglycemia, resulting from defective insulin secretion, impaired cellular insulin response, or a combination of these pathophysiological mechanisms. This disorder underscores a complex interplay of physiological dysfunctions that contribute to its pathogenesis. Diabetes mellitus has undergone progressive global escalation, establishing it as a major global health challenge. Effective management of this condition requires a multidimensional therapeutic strategy combining behavioral modifications, systematic biomarker monitoring, and targeted pharmacological interventions. This multifaceted strategy is essential for maintaining optimal blood glucose levels and preventing complications associated with the disease. Over time, various classes of antidiabetic medications have been developed to target different pathophysiological aspects of the disease.

Diabetes medications can be broadly categorized into oral hypoglycemic agents (OHAs) and injectable therapies. Oral medications serve as the cornerstone for treating T2DM, whereas injectable options, including insulin and glucagon-like peptide-1 receptor agonists (GLP-1 RAs), are implemented for both type 1 diabetes mellitus (T1DM) and more advanced stages of T2DM. This therapeutic stratification reflects the progressive nature of the disease and the need for tailored treatment strategies.

The selection of therapy depends on various factors, including patient-specific characteristics, comorbidities, the risk of hypoglycemia, and cardiovascular outcomes. Recent advances in diabetes pharmacotherapy have emphasized individualized treatment approaches, integrating newer agents with proven benefits beyond glycemic control, such as cardiovascular and renal protection [16].

Given the inconsistent findings across populations, this study aimed to evaluate whether metformin use is associated with a reduced risk of dementia in Taiwanese patients with type 2 diabetes.

We hypothesized that metformin users would exhibit a lower incidence of dementia compared to patients using other antidiabetic medications.

## 2. Materials and Methods

### 2.1. Materials and Participants

**Figure 1:**
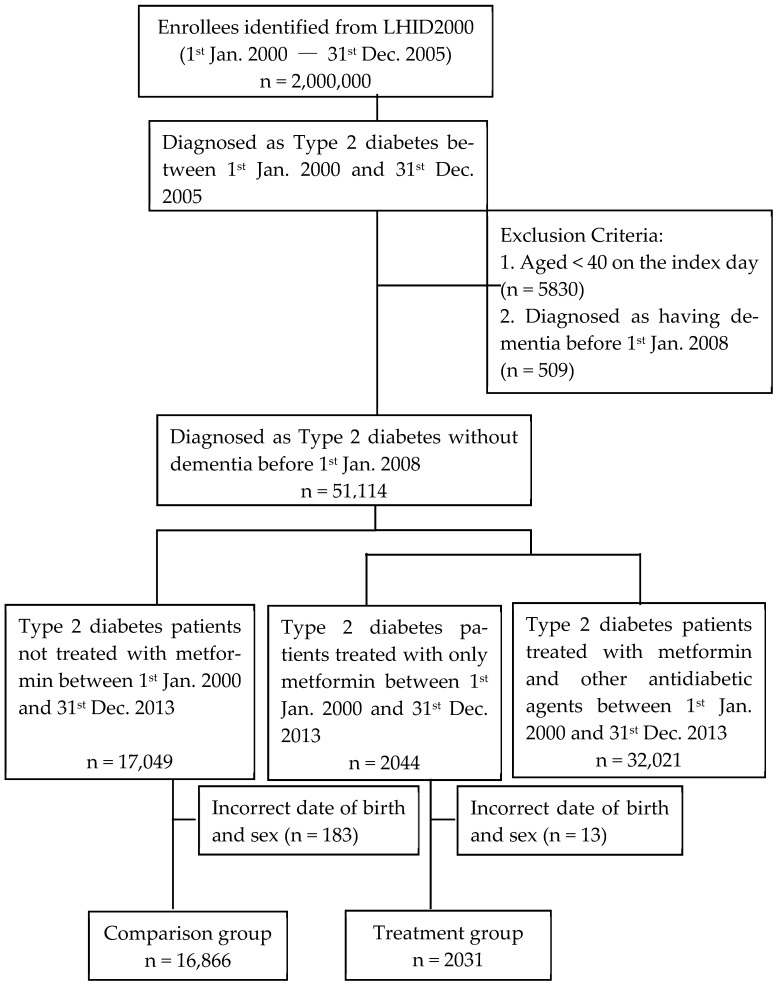
Flowchart of enrolment of participants.

This study used the sub dataset of the NHIRD, utilizing a population-based sampling strategy; the study cohort comprised two million participants selected through probabilistic sampling methods during the 2000–2013 epidemiological observation window. The research employed a retrospective cohort study design. Since its inception in 1995, Taiwan’s resident enrollment rate has consistently exceeded 99%. To maintain data accuracy, regular expert reviews were conducted (Figure 1).

### 2.2. Research Design

The study defined cases of T2DM using the International Classification of Diseases, 9th Revision, Clinical Modification (ICD-9-CM) codes 250.0, 250.1, 250.2, 250.3, 250.4, 250.5, 250.6, 250.7, 250.8, and 250.9, combined with the use of antidiabetic medications. Within the defined cases of T2DM, patients who initiated treatment with metformin (ATC code: A10AB02) between 1 January 2000 and 31 December 2005 were defined as the exposed cohort. In contrast, patients with T2DM receiving second-line treatment for diabetes between 1 January 2000 and 31 December 2005, including insulin (ATC code: A10AB01), sulfonylureas (ATC code: A10BB), DPP-4 inhibitors (ATC code: A10BH01), meglitinides (ATC code: A10BX), thiazolidinediones (ATC code: A10BG), and alpha-glucosidase inhibitors (ATC code: A10BF), were treated as the unexposed cohort. Under this definition, individuals in the exposed cohort were required to have used metformin exclusively during the follow-up period, while those in the unexposed cohort had no record of metformin use at any point during the follow-up. The date of initiating anti-diabetes treatment was designated as the index date. The duration of the event was defined as the time (in years) from the index date to the last recorded prescription between 1 January 2008 and 31 December 2013. Both groups were followed until 31 December 2013. The incidence of dementia was the outcome.

### 2.3. Study Cohort

The investigation enrolled 51,114 treatment-naïve T2DM patients initiating antidiabetic pharmacotherapy between 1 January 2000 and 31 December 2005. Exclusion criteria comprised the following: (1) age < 40 years at treatment commencement, (2) mortality events preceding 1 January 2008, and (3) pre-existing dementia diagnoses documented prior to cohort entry.

### 2.4. Bias Control

Protopathic bias: To mitigate protopathic bias that could result from delayed dementia diagnosis, a 2-year lag-time was implemented, and therefore those with incident dementia from 1 January 2006 to 31 December 2007 were excluded.

Confounders: This study used a logistic regression model with covariates of age, gender, index date, and comorbidities to calculate the propensity score. The exposed and the unexposed cohorts were matched in a 1:4 ratio using propensity scores to control for the aforementioned covariates as confounding factors.

### 2.5. Outcome Variable

The primary analytical outcome was dementia incidence density (ICD-9-CM: 290, 291.2, 292.82, 294.1, 294.2, 331.1, 331.82) in pharmacotherapy-exposed and comparator cohorts during the 2008–2013 surveillance period. Case adjudication required ≥3 dementia-specific outpatient encounters, with previous validation studies confirming 90% diagnostic accuracy for ICD-9-coded dementia in this population [17]. Cohort follow-up extended from index date (1 January 2008) until dementia diagnosis, all-cause mortality (ascertained through National Health Insurance deregistration records), or study termination (31 December 2013). Person-years analysis served as the denominator for dementia incidence density estimation.

### 2.6. Comorbidities

This study defined comorbidities for both the exposed and unexposed cohorts as cases diagnosed with hypertension (ICD-9-CM codes 401–405), hyperlipidemia (ICD-9-CM code 272.4), cardiovascular diseases (ICD-9-CM codes 410–414, 425, 428, 674, 678), chronic kidney disease (ICD-9-CM code 585), and malignant neoplasms (ICD-9-CM codes 140–239) during the period from 1 January 2000 to 31 December 2005. These conditions are common comorbidities among patients with type 2 diabetes and are also known to increase the risk of developing dementia. Therefore, they were included as important covariates in our analysis.

### 2.7. Statistical Analysis

The distributions of demographic characteristics and comorbidities between the exposed and unexposed cohorts were tested for differences using the chi-square test for categorical variables. This study utilized logistic regression to compute propensity scores, with variables of age, gender, index date, and comorbidities. To reduce confounding bias, a greedy nearest-neighbor propensity score matching algorithm with a 1:4 ratio was applied. Matching was performed on age, sex, and comorbid conditions to improve baseline comparability between the metformin-exposed and non-exposed groups. Cox proportional hazard regression was employed to calculate the hazard ratio (HR) with a 95% confidence interval (CI) to estimate the association between the use of metformin and the risk of dementia. The significance level for defining differences was set at 0.05. Analyses were performed in SAS v9.4 (SAS Institute, Inc., Cary, NC, USA).

## 3. Results

### 3.1. Population Characteristics

As shown in Table 1, a total of 18,897 patients, including 16,866 in the unexposed cohort (non-use of metformin) and 2031 in the exposed cohort (use of metformin), were included in data analyses. There was no significant difference in age between the control and treatment groups (*p* = 0.938). However, the gender distribution between the groups was significantly different (*p* = 0.004), with a lower proportion of females in the treatment group. In addition to gender and age, five comorbidities were analyzed. The differences in the prevalence of comorbidities between the control and treatment groups were statistically significant. Comparative analysis of five comorbid conditions revealed a distinct epidemiological profile between cohorts: the therapeutic cohort exhibited a reduced burden of chronic kidney disease (CKD) relative to the comparator cohort, whereas all other concomitant conditions demonstrated elevated prevalence rates. Hypertension displayed the most pronounced intergroup disparity, followed sequentially by cardiovascular disorders, diabetic retinopathy, and hyperlipidemia.

To minimize intergroup variability, propensity scores were computed using logistic regression modeling, with chronological age, biological sex, and comorbid conditions included as adjustment variables. The treatment and control cohorts were subsequently matched based on these propensity scores, with detailed results presented in Supplementary Table S1. Following matching, all measured covariates demonstrated balanced distributions between groups. This matching procedure substantially attenuated covariate-related bias in the analytical outcomes relative to the pre-matched dataset.

### 3.2. Dementia Risk

Over a 14-year longitudinal surveillance encompassing 9484 participants, 288 incident dementia cases were identified. Demographic stratification revealed 255 cases within the comparator cohort versus 33 cases in the pharmacotherapy-exposed cohort. Compared to the unexposed cohort, the hazard ratio of developing dementia in the exposed cohort (metformin users) was 0.472 (95% CI: 0.328–0.679, Table 2).

Furthermore, stratified analyses based on age and gender revealed that metformin users exhibited a lower risk of dementia across all age and gender groups. In the gender-stratified analysis, the hazard ratio (HR) was lower in males (HR = 0.318, 95% CI: 0.167–0.608) compared to females (HR = 0.602, 95% CI: 0.387–0.937). Similarly, in the age-stratified analysis, younger age groups exhibited lower HR values (Table 3).

Longitudinal analysis revealed a monotonic inverse association between cumulative metformin exposure duration and dementia incidence, with risk estimates demonstrating progressive attenuation across ascending therapeutic duration quartiles. In the group with a treatment duration of 10–14 years, the hazard ratio (HR) was as low as 0.218 (95% CI: 0.079–0.598) (Table 4).

## 4. Discussion

### 4.1. Main Findings

In this cohort study of diabetic patients in Taiwan, we investigated the protective effect of the diabetes medication metformin against the development of dementia. Our study findings corroborate previous studies [18,19,20,21], confirming that the use of metformin can reduce the risk of dementia. In addition, the decreased risk of dementia associated with metformin use was consistently observed in two genders and different age groups. Furthermore, the risk of dementia was consistently decreased with the longer duration of metformin use. These findings highlight metformin’s role in dementia risk reduction, supporting its potential as both an antihyperglycemic and a neuroprotective agent.

### 4.2. Comparisons with Previous Studies

Accumulating preclinical evidence has proposed that metformin may confer neuroprotection against dementia through multiple pleiotropic mechanisms. Its primary pharmacodynamic action is believed to involve activation of the AMP-activated protein kinase (AMPK) pathway, which enhances insulin sensitivity and improves peripheral insulin resistance in patients with T2DM—an axis potentially critical to maintaining cerebral glucose homeostasis. Additionally, metformin has been shown in experimental models to exhibit anti-inflammatory effects by downregulating the NF-κB pathway, thereby attenuating neuroinflammatory cascades and possibly reducing pro-inflammatory neurodegeneration.

Metformin is also hypothesized to modulate oxidative stress by scavenging reactive oxygen species (ROS) and enhancing endogenous antioxidant defenses, potentially limiting free radical-induced neuronal injury. Furthermore, cerebrovascular protective effects have been suggested, including increased endothelial nitric oxide bioavailability and reduced atherogenesis, which may help mitigate the risk of vascular dementia [22]. More recently, remodeling of the gut microbiota has been explored as a novel therapeutic axis, with emerging evidence indicating that metformin may influence microbial-derived metabolites that contribute to cognitive maintenance via gut–brain axis signaling [23].

It is important to emphasize, however, that while these mechanisms are biologically plausible and supported by preliminary findings, they remain speculative and have not been conclusively established in human studies. Further mechanistic and longitudinal research is required to validate these proposed pathways.

Because over 99% of Taiwanese patients with T2DM receive treatment, this study was unable to establish a “clean comparison group” as in other studies [18]. This study explored the effects of the duration of continuous metformin use during the follow-up period. Longitudinal analysis demonstrated a temporal-dependent neuroprotective association, with sustained metformin administration exhibiting progressive dementia risk attenuation. This observation further supports the potential of using this widely available medication for dementia prevention. Additionally, previous studies conducted in the U.S. with veterans suggested that the protective effect of metformin was only significant in populations younger than 75 years [19,20]. However, the results of the present study indicate that, in the Taiwanese population, individuals aged 55 and above exhibited significant effects. This phenomenon may be explained by the possibility that the protective effect of metformin on dementia is influenced by ethnic differences, but further research with more data is needed to explore this hypothesis in depth. In a study conducted in South Korea, metformin treatment showed no association with changes in the AD component scores or the activities of daily living index. Notably, the metformin-treated cohort exhibited measurable cognitive deterioration, quantified through Mini-Mental State Examination (MMSE) assessments and impaired immediate verbal recall performance relative to baseline neurocognitive metrics [24]. This suggests that local factors such as dietary habits, healthcare quality, and economic conditions may influence the risk of dementia. Therefore, the conclusions drawn from this study may be specific to the Taiwanese population.

### 4.3. Strengths

The strengths of this study include the use of the NHIRD, which covers more than 99% of Taiwan’s population. Since the LHID 2000 is a database of 1 million subjects randomly selected from the NHIRD, the sample is representative of the general population of Taiwan. As NHIRD is primarily used for administrative billing purposes, the prescription data is both accurate and reliable. Additionally, the large sample size and extended follow-up period further enhance the robustness of this study. Additionally, propensity score matching was applied before statistical analysis to reduce demographic and comorbidity differences between the cohorts.

## 5. Limitations

This study used data from the NHIRD for analysis. However, the database primarily focuses on insurance and financial aspects, with incomplete information on healthcare-related data, which is an inherent limitation of the database. As a result, we were only able to track patients’ medication records but had no access to detailed information about their medication adherence. Additionally, data regarding patients’ lifestyle factors, such as exercise habits, smoking, and alcohol consumption, were not available. Furthermore, for operational reasons, this study classified all patients who were taking other antidiabetic medications (such as sulfonylureas, insulin) into the comparison group. The association between these medications and dementia was not addressed in this analysis. Additionally, this study included all type 2 diabetes patients using other medications as the comparison group. These patients may use one or more anti-diabetic drugs, whose effects on dementia remain uncontrolled. Moreover, potential interactions from multiple drug use could complicate outcomes. Addressing these uncertainties requires larger datasets and more in-depth investigation.

Another potential limitation of our study is that in Taiwan, metformin is typically prescribed to patients with preserved renal function and without a history of severe cardiovascular or hepatic diseases. This prescribing pattern may introduce a healthy user bias, potentially leading to a spurious protective association between metformin use and reduced dementia risk. Healthier individuals, by virtue of better overall physiological reserve and fewer comorbidities, are generally at lower risk of developing dementia, regardless of pharmacologic exposure. Therefore, the observed association might partly reflect underlying differences in baseline health rather than a true drug effect. However, we attempted to mitigate this confounding effect by incorporating major comorbidities into our propensity score matching model, which may have partially adjusted for differences in baseline health status between the groups.

## 6. Conclusions

Our findings suggest that metformin use may be associated with a lower risk of developing dementia in individuals with type 2 diabetes mellitus, though further research is needed to confirm causality. As the global burden of diabetes continues to rise, with projections indicating that by 2045, 548 million people will be affected, it is crucial to explore effective interventions not only for diabetes management but also for reducing the risk of dementia. The growing body of evidence linking diabetes and Alzheimer’s highlights the need for further research to optimize diabetes treatment strategies and address their potential role in preventing or delaying AD onset.

## Figures and Tables

**Table 1:** Characteristics of study subjects with and without medication treatment.

Variables	Comparison Group (Non-Use of Metformin)		Treatment Group (Use of Metformin)		*p*-Value
	(n = 16,866)		(n = 2031)		
	Mean/No.	SD/%	Mean/No.	SD/%	
Age	58.499	10.936	58.479	11.051	0.938
Gender					
Females	8991	53.3	1042	51.3	0.004 *
Males	7875	46.7	988	48.7	
Hypertension					
NO	10,829	64.2	1033	50.9	<0.0001 *
YES	6037	35.8	998	49.1	
Hyperlipidemia					
NO	12,719	75.4	1426	70.2	<0.0001 *
YES	4147	24.6	605	29.8	
CVD					
NO	15,071	89.4	1745	85.9	<0.0001 *
YES	1795	10.6	286	14.1	
CKD					
NO	16,492	97.8	2011	99.0	<0.0001 *
YES	374	2.2	20	1.0	
MT					
NO	16,512	97.9	1972	97.1	0.019 *
YES	354	2.1	59	2.9	

Abbreviations: CVD, cardiovascular disease; CKD, chronic kidney disease; MT, malignant tumor; SD, standard deviation. * Indicates a statistically significant difference as defined in this study.

**Table 2:** Association of use of metformin with dementia risk.

Group	No. of Subjects	No. of Dementia Cases	HR	95% CI	*p*-Value
Non-Users	7484	255			
Users	2000	33	0.472	0.328–0.679	<0.0001 *

Abbreviations: HR, hazard ratio; CI, confidence interval. Hazard ratios were adjusted for age, gender, and the presence of hypertension, hyperlipidemia, cardiovascular disease, chronic kidney disease, and malignant tumor. * Indicates a statistically significant difference as defined in this study.

**Table 3:** Stratified analysis of the association between use of metformin and dementia risk on the basis of gender and age.

Group	No. of Dementia Cases	No. of Subjects	HR	95% CI	*p*-Value
Gender					
Males	10	977	0.318	(0.167–0.608)	0.001 *
Females	23	1023	0.602	(0.387–0.937)	0.024 *
Age (year)					
40–55	2	851	0.325	(0.77–1.376)	0.127
55–70	13	804	0.452	(0.255–0.802)	0.007 *
>70	18	345	0.537	(0.327–0.882)	0.014 *

Hazard ratios were adjusted for the presence of hypertension, hyperlipidemia, cardiovascular disease, chronic kidney disease, and malignant tumor, and age (for gender effect) or gender (for age effect). * Indicates a statistically significant difference as defined in this study.

**Table 4:** Association between the duration of metformin use and dementia risk.

Group	No. of Dementia Cases	No. of Subjects	HR	95% CI	*p*-Value
Duration time (years)					
2–6	9	159	0.863	(0.420–1.772)	0.688
6–10	20	982	0.475	(0.297–0.759)	0.002 *
10–14	4	859	0.218	(0.079–0.598)	0.003 *

Hazard ratios were adjusted for age, gender, and the presence of hypertension, hyperlipidemia, cardiovascular disease, chronic kidney disease, and malignant tumor. * Indicates a statistically significant difference as defined in this study.

## Data Availability

The original contributions presented in this study are included in the article. Further inquiries can be directed at the corresponding author.

## Supplementary Materials

The following supporting information can be downloaded at: https://www.mdpi.com/article/10.3390/healthcare13131537/s1, Table S1. Characteristics of study subjects with and without drug after propensity score matching.
