# Supplementary material for: Association of Metformin with the Risk of Dementia: A Population-Based Retrospective Cohort Study in Taiwan
Source: Healthcare (Basel). 2025 Jun 27;13(13):1537. doi: 10.3390/healthcare13131537 (PMC12249156; doi:10.3390/healthcare13131537)
Supplement: Supplementary file 1 [file healthcare-13-01537-s001.zip › healthcare-3600520-supplementary.pdf]

Supplementary Materials

Table S1. Characteristics of study subjects with and without drug after propensity score matching

Variables	Comparison group (Non-use of metformin) (n=7484)		Treatment group (Use of metformin) (n=2000)		*P*-value
	Mean/No.	SD/%	Mean/No.	SD/%	
Age	57.724	10.723	57.934	10.935	0.438
Gender					
Female	3816	51.0	1023	51.2	0.898
Male	3668	49.0	977	48.8	
Hypertension					
NO	4074	54.4	1023	51.1	0.009*
YES	3410	45.6	977	48.9	
Hyperlipidemia					
NO	5378	73.9	1417	70.8	0.373
YES	2106	28.1	583	29.2	
CVD					
NO	6553	87.6	1720	86.0	0.063
YES	931	12.4	280	14.0	
CKD					
NO	7437	99.4	1987	99.4	0.912
YES	47	0.6	13	0.7	
MT					
NO	7308	97.6	1946	97.3	0.368
YES	176	2.4	54	2.7	

Propensity scores were calculated using logistic regression with covariates of age, gender, and comorbidities.
